# Refractory Duodenal Ulcer in a Patient With Median Arcuate Ligament Compression: A Treatment Challenge

**DOI:** 10.14309/crj.0000000000001407

**Published:** 2024-08-05

**Authors:** Sérgio Manuel Tubal Bronze, Daniel Conceição, Milena Mendes, Filipe Cardoso, Daniel Torres, Elia Coimbra, Tiago Bilhim

**Affiliations:** 1 Serviço de Gastrenterologia e Hepatologia, Centro Hospitalar Universitário Lisboa Norte, Lisboa, Portugal; 2 Faculdade de Medicina de Lisboa, Universidade de Lisboa, Lisboa, Portugal; 3 Serviço de Gastrenterologia, Instituto Português de Oncologia Gentil Martins, Lisboa, Portugal; 4 Serviço de Gastrenterologia, Centro Hospitalar Universitário Lisboa Central, Lisboa, Portugal; 5 Unidade de Transplantes, Centro Hospitalar Universitário Lisboa Central, Lisboa, Portugal; 6 Unidade de Radiologia de Intervenção, Centro Hospitalar Universitário Lisboa Central, Lisboa, Portugal; 7 NOVA Medical School, Faculdade de Ciências Médicas, Universidade Nova de Lisboa, Lisboa, Portugal

**Keywords:** gastrointestinal bleeding, arterial embolization, median arcuate ligament

## Abstract

Flexible esophagogastroduodenoscopy is the gold standard for the management of acute upper gastrointestinal bleeding. This is a case of a man who was admitted in the emergency department because of melena with hypotension because of an ulcer in the anterior face of the duodenal bulb, refractory to 3 attempts of endoscopic therapy. Then, a gastroduodenal arterial embolization was tried, being impossible because of the presence of the median arcuate ligament, compressing the celiac trunk. A balloon-expandable stent was inserted in the celiac trunk, and then, the embolization was performed. After unsuccessful endoscopic management, the arterial embolization is one of the treatment options in nonvariceal acute upper gastrointestinal bleeding.

## INTRODUCTION

Flexible esophagogastroduodenoscopy is the gold standard for the management of acute upper gastrointestinal bleeding.^1^ In those patients whose endoscopic management fails or in those with hemodynamic instability, the endovascular approach provided by interventional radiology is indicated.^1^

## CASE REPORT

**Figure 1.**
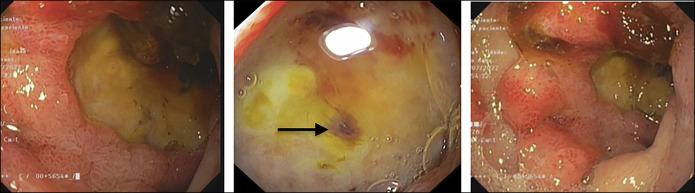
First endoscopy - posterior wall of bulb with an excavated ulcer with flat pigmented spot (black arrow) (Forrest IIc).

**Figure 2.**
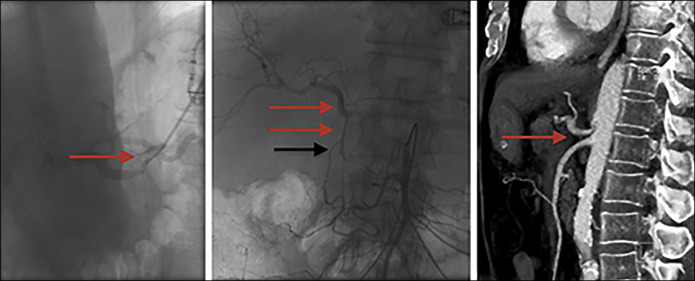
Lateral view selective catheter angiography (left image) confirming a tight stenosis of the celiac trunk origin (arrow). Neutral view catheter angiography of the superior mesenteric artery (middle image) depicting retrograde filling of the gastroduodenal artery and hepatic artery (black arrow). Also note a small pseudoaneurysm in the distal part of the gastroduodenal artery (dotted arrow). Celiac trunk stenosis (red arrow) depicted in CT angiography sagittal reformat (right image).

**Figure 3.**
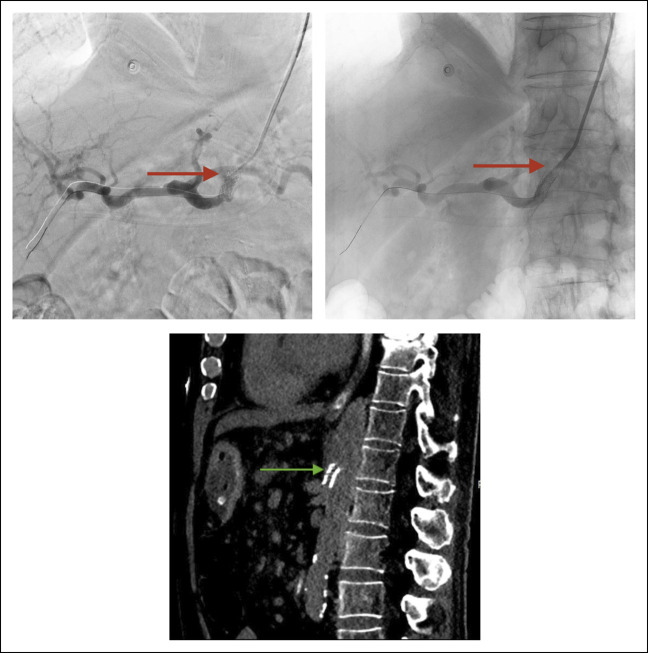
Control angiography after stent placement in the celiac trunk stenosis (red arrows) with (left side image) and without (right side image) bone subtraction. Intra-procedural CT was performed with sagittal reformat (bottom image) to confirm correct stent placement covering the celiac trunk stenosis into the aorta.

**Figure 4.**
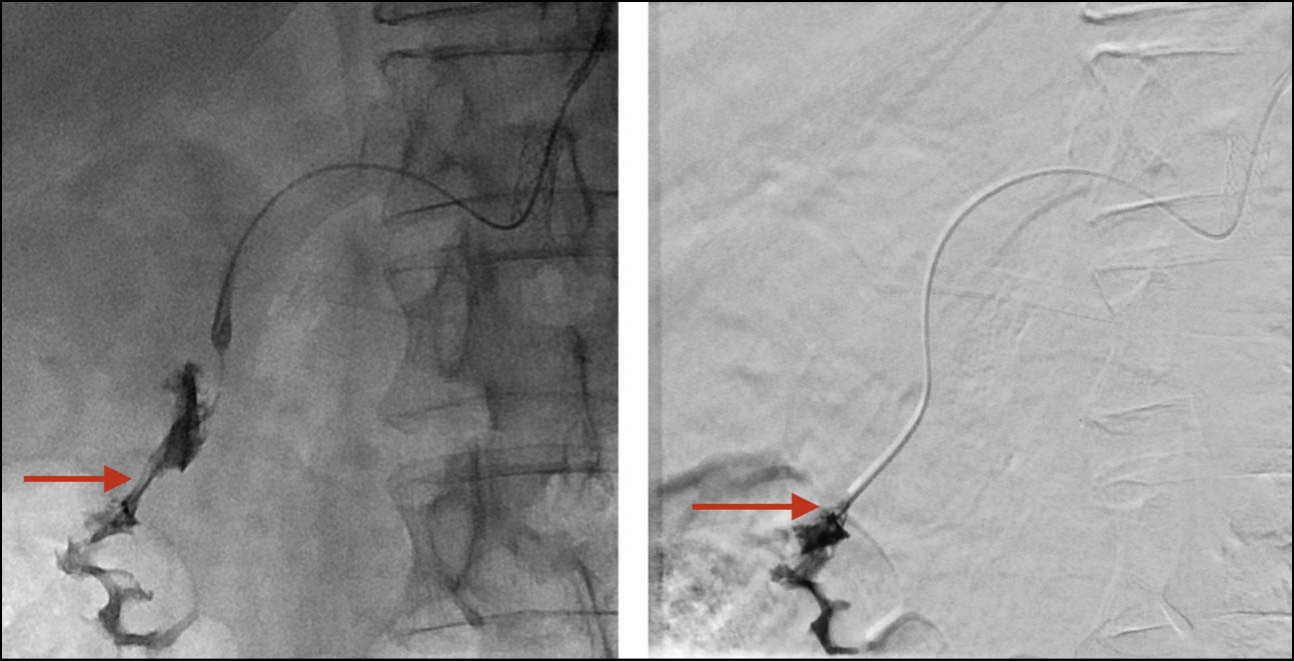
Arterial-duodenal fistula seen after the injection of contrast in the gastroduodenal artery with extravasation to the duodenum (red arrow).

**Figure 5.**
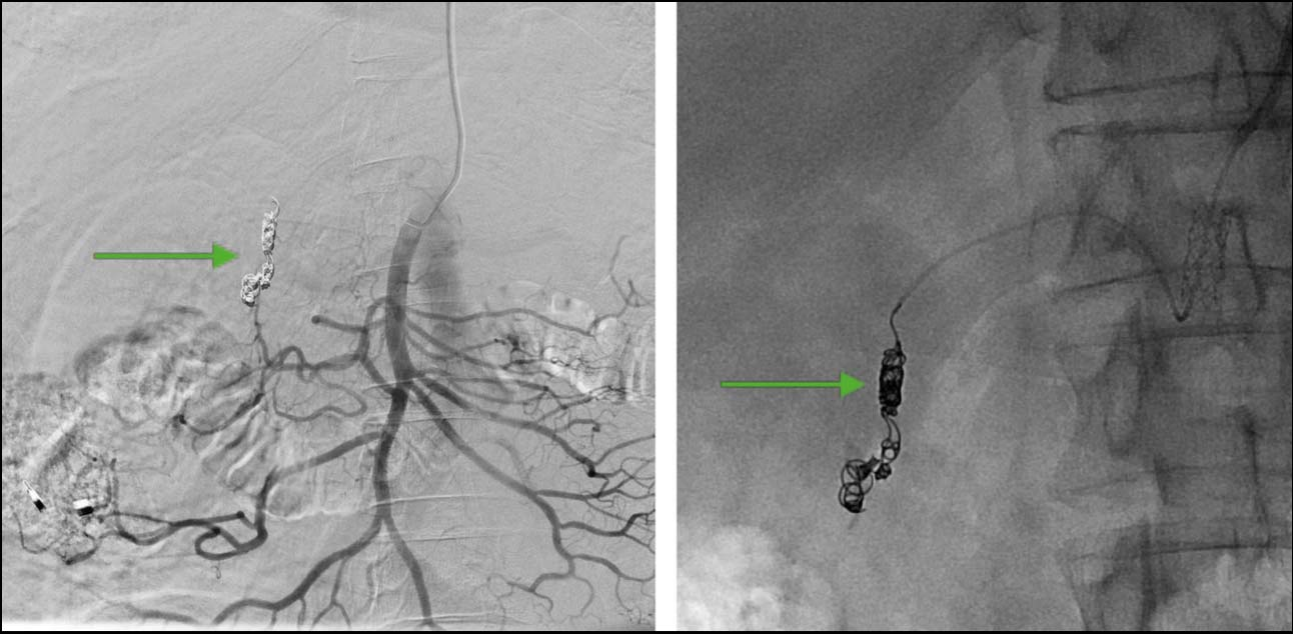
Coils (green arrows) placed in the gastroduodenal artery. Final control angiogram of the superior mesenteric artery confirming the occlusion of the gastroduodenal artery with no retrograde filling of the celiac trunk (left side image). Fluoroscopy image while deploying the coils in the gastroduodenal artery (right side image).

We report a 70-year-old man with a medical history of arterial hypertension under candesartan, with no known allergies. He had alcohol intake that could not be specified. He was admitted to the emergency department with the chief complaint of melena lasting for the past 3 days. In the admission, he was disorientated with hypotension (systolic blood pressure 98 mm Hg). First blood workup showed hemoglobin 5.7 g/dL, platelets 223,000/L, prothrombin time 14.3 seconds (international normalized ratio 1.19), urea 137 mg/dL, creatinine 1.59 mg/dL, aspartate aminotransferase 41 U/L, and C-reactive protein 19.9 mg/L. Arterial gasometry showed normal pH and a lactate of 4.8 mmol/L. Fluid challenge was started, and 2 red blood cells' concentrates were given. Soon after stabilization, he went through an upper endoscopy that showed on the anterior face of the duodenal bulb, a 20-mm excavated ulcer with a flat pigmented spot (Forrest IIc), the reason why it was not endoscopically treated (Figure 1). Two days later, the patient had an episode of hematochezia without hemodynamic instability, and a second upper endoscopy was performed, revealing the same findings. The gastroenterology team tried to attach 2 clips through the scope, which failed, and then injected adrenaline and polidocanol, ending the procedure with the application of hemospray, achieving immediate hemostasis. Attending clinical stability, he was then transferred to the general surgery ward. On the second day after the admission to the ward, the patient had an episode of melena associated with hypotension and mental status alteration (Glasgow coma scale of 3), and soon after stabilization with fluids, red blood cells, and orotracheal intubation, he was transferred to the intensive care unit. Hemoglobin at this time was 6.3 g/dL, and after the transfusion raised to 8.8 g/dL, no thrombocytopenia or prolongation of prothrombin time was seen. The gastroenterologist team gave their opinion on whether the patient should undergo a third endoscopy, and the decision was that, according to the hemorrhagic recidive, endovascular or surgical approaches should be tried. The interventional radiology team agreed on the gastroduodenal embolization, and a first attempt was made. The celiac trunk angiography showed flux inversion in the gastroduodenal arcade secondary to a celiac trunk stenosis of approximately 14 mm, findings confirmed after superior mesenteric angiography (Figure 2). An 5-mm aneurysm was also identified in the gastroduodenal artery. The stenosis was also confirmed with a computed tomography, caused by the presence of the median arcuate ligament, compressing the celiac trunk. The radiology team tried to put a stent without success through the femoral artery, and then, a second approach was made through the radial artery. After super-selective catheterization of the common hepatic artery, a balloon-expandable stent with 7 × 18 mm was inserted in the celiac trunk (Figure 3). The angiographic and computed tomography control showed resolution of the stenosis (Figure 3). The catheterization of the gastroduodenal artery confirmed an arterial-duodenal fistula (Figure 4). The embolization was performed with 5 coils of 5 mm and 1 coil of 7 mm, with exclusion of the gastroduodenal artery and exclusion of the fistula as major outcomes (Figure 5). No more episodes of gastrointestinal bleeding were noticed with further hemoglobin level stable. The patient continued in the intensive care unit because of other problems triggered by the hypovolemic shock, such as neurologic dysfunction and acute kidney injury. One week after the procedure, an esophagogastroduodenoscopy was performed, and the ulcer reduced its dimensions with no identifiable high-risk stigmata for rebleeding.

## DISCUSSION

After unsuccessful endoscopic management, the arterial embolization is one of the treatment options in nonvariceal acute upper gastrointestinal bleeding.^1^ This report shows a challenging case in a patient with median arcuate ligament compression, needing a 2-step procedure, first putting a stent in the celiac trunk followed by gastroduodenal artery embolization. When pain or other typical features such as vomiting or weight loss are not present in the compression of the celiac trunk by the arcuate ligament, it acquires the name of median arcuate ligament compression instead of arcuate ligament syndrome or Dunbar syndrome. This compression occurs in 10%–25% of the population.^2^ This stepwise treatment has been previously described for pancreaticoduodenal and gastroduodenal artery aneurysms associated with celiac artery compression.^3^ The association of nonvariceal acute upper gastrointestinal bleeding because of gastroduodenal artery pseudoaneurysm in a patient with a median arcuate ligament has also been described with selective embolization of the aneurysm.^4^ There is a proven risk of visceral ischemia if embolization of the gastroduodenal artery is performed without revascularization of the celiac trunk.^5^ Aneurysms of the gastroduodenal and pancreaticoduodenal arteries are frequently associated with celiac trunk stenosis or occlusions and should be treated by an endovascular-first approach because of the high risk of rupture.^6^ In this patient, the small size of the aneurysm and the large and deep ulcer with close contact to the gastroduodenal artery might imply that the aneurysm was due to the duodenal ulcer and not secondary to the median arcuate ligament compression. This hypothesis is further supported by the fact that an arterioduodenal fistula was confirmed after minimal catheterization of the gastroduodenal artery.

## DISCLOSURES

Author contributions: SMT Bronze coordinated paper writing and is the article guarantor. D. Conceição contributed to manuscript writing. M. Mendes, F. Cardoso, D. Torres, and E. Coimbra contributed to manuscript revision. T. Bilhim contributed to paper writing and its critical revision.

Financial disclosure: None to report.

Informed consent was obtained for this case report.
